# Evaluating the influence of environmental variables on the length-weight relationship and prediction modelling in flathead grey mullet, *Mugil cephalus* Linnaeus, 1758

**DOI:** 10.7717/peerj.14884

**Published:** 2023-02-24

**Authors:** Rejani Chandran, Rajeev K Singh, Achal Singh, Kantharajan Ganesan, Ajith Kumar Thipramalai Thangappan, Kuldeep K Lal, Vindhya Mohindra

**Affiliations:** 1 Fish Conservation Division, ICAR-National Bureau of Fish Genetic Resources, Lucknow, Uttar Pradesh, India; 2 PMFGR Centre, ICAR-National Bureau of Fish Genetic Resources, Kochi, Kerala, India; 3 ICAR-Central Institute of Brackishwater Aquaculture (CIBA), Chennai, Tamil Nadu, India

**Keywords:** Regression coefficient, Condition factor, Fitness, Conservation, Management, Biology, Mariculture

## Abstract

Fish stocks that are grown under diverse environmental conditions have different biometric relationships and growth patterns. The biometric length-weight relationship (LWR) is an essential fishery assessment tool, as fish growth is continuous and depends on genetic and environmental factors. The present study attempts to understand the LWR of the flathead grey mullet, *Mugil cephalus* Linnaeus, 1758, from different locations. The study area encompassed its distribution in the wild across freshwater location (one), coastal habitats (eight locations), and estuaries (six locations) in India to determine the relationship between various environmental parameters. Specimens (*n* = 476) of *M. cephalus* were collected from commercial catches and the length and weight of individual specimens were recorded. Monthly data from the study locations were extracted for nine environmental variables from the datasets downloaded from the Physical Oceanography Distributed Active Archive Center (PO.DAAC) and the Copernicus Marine Environment Monitoring Service (CMEMS) over 16 years (2002 to 2017) on the Geographical Information System platform. The parameters of the LWR, intercept ‘a’ and slope or regression coefficient ‘b’, varied from 0.005321 to 0.22182 and 2.235 to 3.173, respectively. The condition factor ranged from 0.92 to 1.41. The partial least squares (PLS) score scatter plot matrix indicated differences in the environmental variables between the locations. PLS analysis of the regression coefficient and environment parameters revealed that certain environment variables *viz.*, sea surface temperature, salinity, dissolved oxygen, nitrate, and phosphate, played a positive role. However, chlorophyll, pH, silicate, and iron played a negative role in influencing weight growth across various locations. The results revealed that the *M. cephalus* specimens from three locations, Mandapam, Karwar, and Ratnagiri, possessed significantly higher fitness to their environment than those from the other six locations. The PLS model can be used to predict weight growth under the various environmental conditions of different ecosystems. The three identified locations are useful sites for the mariculture of this species considering their growth performance, the environmental variables, and their interactions. The results of this study will improve the management and conservation of exploited stocks in regions affected by climate change. Our results will also aid in making environment clearance decisions for coastal development projects and will improve the efficiency of mariculture systems.

## Introduction

The length-weight relationship (LWR) is one of the most critical functional parameters used to assess exploited fishery stocks to understand a species’ fitness in their environment (Nazek et al., 2018). Fish stocks growing under diverse environmental conditions are likely to show different growth rates, while within a stock, differences in LWR between males, females, juveniles, and adults may also be exhibited (Prasad & Rao, 2015). The LWR is used for predicting the weight corresponding to a given length and thus allows for the comparison of fish growth between different regions or localities (Tsoumani et al., 2006). LWR studies also play a vital role in predicting a fish population’s feeding intensity, metamorphosis, and general well-being (Ahmad et al., 2012). With knowledge of this relationship, one may determine the number of fish landed, measure populations over space and time (Khan et al., 2011), calculate the catch in terms of weight or biomass, compare the inter-specific and inter-population morphometric composition of fish species, and assess the general well-being of the fish population (Hajjej et al., 2011). The condition factor compares the condition, fatness, or well-being of the fish and provides information on food abundance and duration of breeding (Ao, Omolara & Eteobong, 2017), which is useful when comparing two populations in different regions, climates, and other conditions (Wheatherly & Gill, 1987). The differences in the ‘b’ value between locations imply that the role of environment variables may be balancing in nature. However, some locations have more favorable fish growth patterns compared to others. Indian coastal waters are strongly influenced by monsoons and other associated events, which cause an enormous influx of freshwater (with varying physicochemical properties) along the Bay of Bengal and the Arabian Sea (Pradhan, Shirodkar & Sahu, 2009; Srinivasan, Natesan & Parthasarathy, 2013; Naik et al., 2020). This causes a marked difference in the water quality parameters on a spatial and temporal basis along the coasts. Further, seasonal variation in the temperature also contributes to changes in the water quality parameters (Gupta, Dhage & Kumar, 2009). The partial least squares (PLS) model enables researchers to estimate complex relationships with many constructs, indicator variables, and structural paths without imposing distributional assumptions on the data (Hair et al., 2019). Recently, this model was employed to determine the histamine content in tuna fish samples (Asghari, Hosseini & Ghajarbeygi, 2022), as well as the performance of pigs (Krugmann et al., 2020) and poultry (Gholami et al., 2020) for food production purposes.

The flathead grey mullet, *Mugil cephalus* Linnaeus, 1758, is an economically important euryhaline and eurythermal marine teleost contributing to large fisheries in the shallow coastal waters, lagoons, coastal lakes, and estuaries of India (Rheman et al., 2002). This species has a diverse distribution worldwide between the latitudes 40°N and 40°S. It can be found in all oceans (De Silva, 1980), with the exception of the colder northeast Pacific in the Pacific Province and the Magellanic Province in South America (Barletta & Dantas, 2016). This species has a catadromous migration type. During larval stages they feed on plankton in the marine environment. At a month old they reach the postflexion larval stage and migrate to an estuary where they develop into the juvenile and sub-adult stages, changing from planktonophagous to detritivorous (De Silva, 1980). Adults migrate back to open water to spawn and complete their life cycle. This species is important for roe (female mature gonads) and meat production. It is an excellent candidate species for both mono- and polyculture as it feeds at a lower trophic level on plant detritus and microflora (Odum, 1970). This species is widely cultured as it utilizes both supplemental feed and/or natural food (Lupatsch, Katz & Angel, 2003). Many researchers have carried out aquaculture studies of *M. cephalus* in ponds (Liu et al., 2021), recirculation aquaculture systems (Vinatea et al., 2018), lagoons (Vallainc et al., 2021), and estuarine areas (Pradhan et al., 2020). The culture of this species has been attempted in various parts of India (Pradhan et al., 2020; Kumar et al., 2021) and other countries (Saleh, 2008). In India, the fish is typically reared using the polyculture method from wild collections and is restricted to a traditional system (Abraham et al., 2000) in extensive poly-farming impoundments.

The present study aims to establish the LWR and condition factors of *M. cephalus* from various locations across India, considering this species’ relevance and importance. Efforts were made to consider the possible impact of the environmental parameters of the study sites in the LWR and the well-being of the fish populations. The study aimed to identify potential sites suitable for mariculture in coastal locations across India by considering the environment variables and growth pattern for LWR. PLS was used to describe growth, as assessed through the coefficient of regression ‘b’ and environment variables. This model may help predict the growth performance of *M. cephalus* when subjected to environmental changes.

## Materials and Methods

### Description of the study area

**Figure 1:**
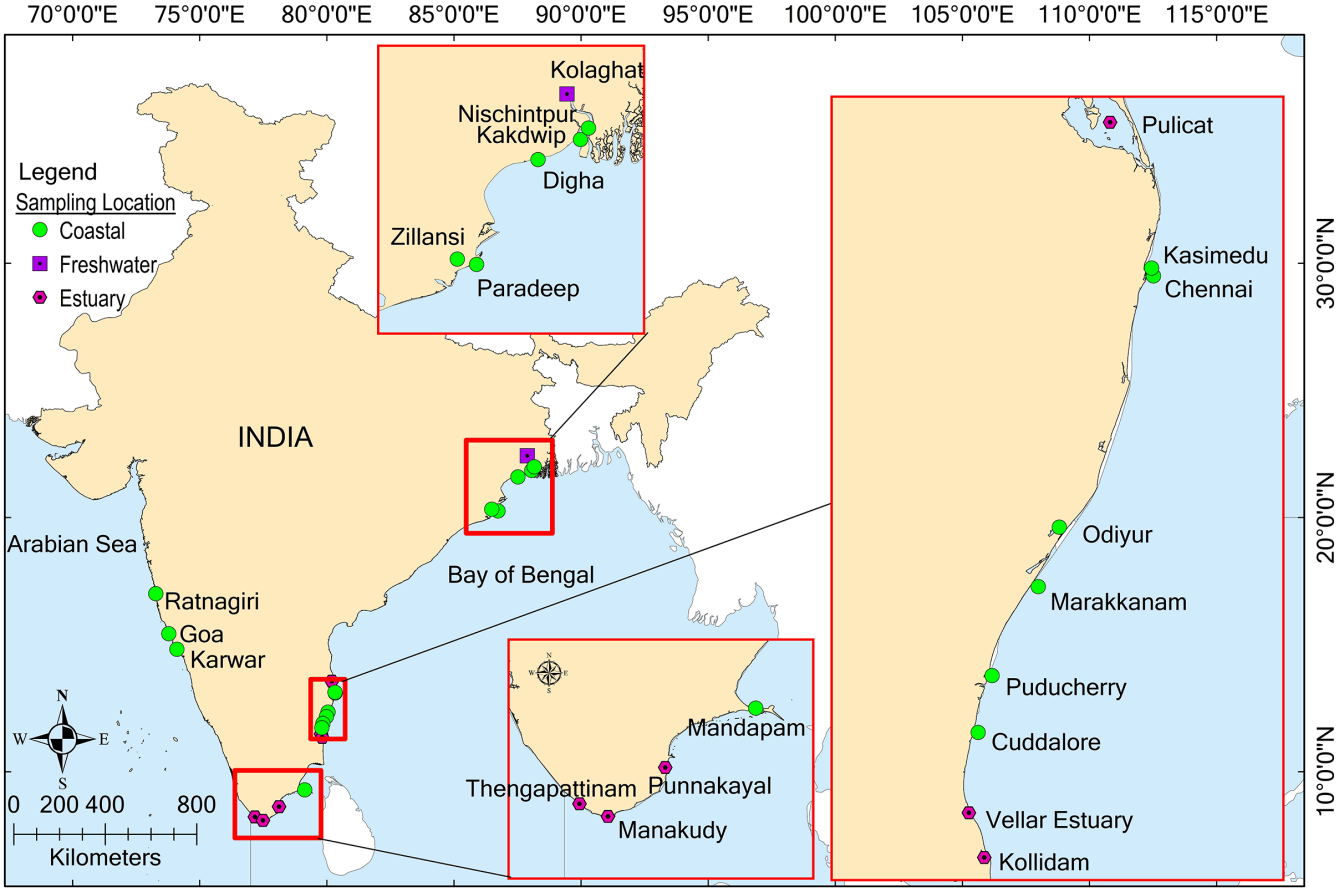
Map depicting the sample collection locations along the Indian coast. Map created using ArcGIS.

The study area was between the 7.5°–23.5°N latitudes and 72°–90°E longitudes, and encompassed diverse ecosystems, including freshwater (one location), coastal habitats (eight), and brackish water (six) across India (Fig. 1). The freshwater sampling site, Kolaghat, is located in the lower stretch of one of the world’s longest rivers, the Ganga. Conscious efforts were made to conduct an extensive sample collection across India, covering the Bay of Bengal (East Coast) and the Arabian Sea (West Coast). Both coasts have notable biological and oceanographical differences in productivity, salinity, wind pattern, monsoon currents, and catchment inflow (Panikkar & Jayaraman, 1966; Kumar et al., 2002). Furthermore, due to the vast geographical extent of the coastline, the regional differences within the coast were reported and included parameters such as, salinity, temperature, river inflow, and fish species composition (Sasamal, 1990; Kumar & Mathew, 1997). Madhupratap et al. (2001) classified the Indian coastal region of the Arabian Sea into northern and southern areas of 15°N latitude, where carnivorous and planktivorous fishes were dominant, respectively. The brackish water sampling locations included the estuaries of a major river (Kollidam–Cauvery River), minor rivers (Vellar–Vellar River, Punnakayal-Thamirabarani River, Manakudy–Pazhayar River, and Thengapattanam-Thamiraparani (Kuzhithuraiar) River) and a coastal lagoon (Pulicat Lake) (ENVIS, 2017) Among these, Thengapattanam is located along the southwest coast, while the other sites are on the southeast coast of India.

### Data collection

#### Length-weight parameters

A total of 476 *M. cephalus* specimens were collected from various locations across the country from 2013 to 2019. These included Kolaghat (19), NE1 inclusive of Nischintpur, Kakdwip, and Digha (38), NE2 inclusive of Paradeep and Zillanasi (27), Chennai inclusive of Chennai and Kasimedu (19), Marakkanam inclusive of Odiyur and Marakkanam (35), Puducherry inclusive of Puducherry and Cuddalore (44), Mandapam (52), SW inclusive of Karwar and Goa (30), Ratnagiri (18), Pulicat Lake (39), Vellar estuary (64), Kollidam (38), Manakudy (34), Thengapattanam (16), and Punnakkayal (13) (Table 1). No live fish were used in the experiments performed. All of the fish were procured from commercial catches and were received already deceased. The length and weight of the individual specimens were recorded accurately to the nearest 0.01 mm and 0.1 g, respectively. The protocols for this study were approved by the Institutional Animal Ethical Committee (IAEC), ICAR-NBFGR, Lucknow, India, vide No. G/CPCSEA/IAEC/2015/2.

**Table 1:** Description of collection localities, number of samples, and size statistics of *Mugil cephalus* in different locations of India.

Sl No	Location	*N*	Year	Code	Lt range (cm)	Wt range (g)
**FRESHWATER**						
1	Kolaghat	19	2013	Kolaghat	15–23.4	33–128
**COASTAL LOCATIONS**						
2	Nischintpur	17	2017	NE1	15–41.5	40–717
	Kakdip	17	2016			
	Digha	4	2015			
3	Paradeep	20	2017	NE2	18.9–49	80–1,179
	Zillanasi	7	2017			
4	Chennai	11	2017	Chennai	22–44.5	112–950
	Kasimedu	8	2013			
5	Odiyur	9	2015	Marakkanam	20–51.2	85–1,421
	Marakkanam	26	2015 (13) 2016 (13)			
6	Puducherry	32	2015 (14) 2016 (18)	Puducherry	19.5–46.2	68–906
	Cuddalore	12	2015			
7	Mandapam	52	2014 (13) 2016 (39)	Mandapam	17–50	53–1,210
8	Karwar	24	2017 (9) 2019 (15)	SW	20–44	58.5–918
	Goa	6	2017			
9	Ratnagiri	18	2017	Ratnagiri	27.3–50.2	280–1,100
**ESTUARY**						
10	Pulicat Lake	39	2017 (25) 2015 (14)	Pulicat lake	32.2–51.2	324–1,421
11	Vellar estuary	64	2015	Vellar	19–46	68–1,075
12	Coleroon estuary	21	2016	Kollidam	14.5–44.5	40–1,000
	Kollidam	17	2015			
13	Manakudy estuary	24	2014 (9) 2016 (15)	Manakudy	15–38.6	40–546
14	Thengapattanam	16	2016	Thengapattanam	18.9–44	80–950
15	Punnakayal	13	2016	Tuticorin	25–48	146–1,017

#### Environmental parameters

The monthly data for various environmental parameters, including salinity, dissolved oxygen (DO), pH, nitrate, phosphate, silicate, iron, and chlorophyll, were obtained from the Copernicus–Marine Environment Monitoring Service (CMEMS) (CMEMS, 2021, 2022a and 2022b); the sea surface temperature (SST) was obtained from the Physical Oceanography Distributed Active Archive Center (PO.DAAC) (NASA-OBPG, 2020). The monthly datasets of all environmental parameters were downloaded and extracted for a period of 16 years (January 2002 to December 2017) on the GIS platform, with the exception of SST (July 2002 to December 2017).

Among the sampling sites (Fig. 1), the proxy locations for environmental data extraction were fixed along the shore for nine coastal locations, including Kakdwip, Paradeep, Chennai, Marakkanam, Puducherry, Cuddalore, Mandapam, Karwar, and Ratnagiri (Table 1). Data extraction was performed using the Spatial Analyst tool in the ArcMap 9.3 platform (ESRI, Redlands, CA, USA). The present study did not consider estuarine and freshwater locations for environmental data recovery due to their ecological characteristics and limitations in pinpointing these locations.

#### Statistical models

Linear regression (LR) analysis of log-transformed length and weight generated parameters for the LWR, such as intercept ‘a’, slope, or regression coefficient ‘b’ (LR) (Snedecor & Cochran, 1967). LWR was calculated using the transformed logarithmic formula of cube law, proposed by Le Cren (1951): Log W = Log a + b Log L where W = weight of fish (g), L = total length of fish (cm). The coefficient of condition factor ‘K’ (Fulton, 1904) was calculated as: K = W * 100/L^3^ where W = weight in grams, L = total length in cm, and 100 is a factor to bring the value of K to near unity. The relative condition factor ‘K_n_’ (Le Cren, 1951), K_n_ = W_t_/W_e_, where W_t_ is observed weight and W_e_ is expected weight, was quantified to understand the health and condition of the fish. All statistical analyses for weight growth and climate data were performed using SPSS version 16 (SPSS Inc. Released, 2007), SAS version 9.3 (SAS Institute Inc., 2012), and Excel (Microsoft Corporation, 2018). The mean of nine environment variables was assessed across the nine locations selected. The estimated weight growth-‘b’ (LR), derived from the linear regression (LR) model of weight (W) and length (L) in the logarithmic scale of *M. cephalus*, was used in association with nine environment variables through the PLS model for the prediction of environment-influenced weight growth-‘b’ (PLS) over nine locations.

The partial least squares model for regression of the response variable by an explanatory variable may be defined as:

${\rm Y = X\; (WQ) + E}$

where

Y: Matrix of the response variable (or weight growth over locations or b(LR)), 9 × 1;

X: Matrix of explanatory variables (environment variables), 9 × 9;

W: Matrix of weight for an environment variable, 9 × 9;

Q: Matrix of regression coefficients (loadings) of Y on X, 9 × 1;

E: Error term.

PLS modelling for regression of weight growth (Y) with environment variables (X) over nine locations generated a covariance matrix (Y′XX′Y). A suitable number of factors in the cross-validation between root mean square error (RMSE) and the predicted residual error sum of squares (PRESS) was identified as measures of criterion. Selected factors (PLS factors) were considered for explaining the variation in the environment variables (X), response variables (Y), scores analysis for Y, X, and distribution of loadings (X-loadings), the association of weightage (X-weights), variables of importance for X (VIP), graphical distribution for the association of ‘b’ (LR), environment variables (X) and ‘b’ (PLS), and also for correspondence between ‘b’ (LR) to ‘b’ (PLS). These results were used to observe the differences in weight from the LWR by regression model and the weight growth influenced by varying environment variables from the PLS model between sampling locations.

## Results

### Length-weight regression and condition factor analysis

**Figure 2:**
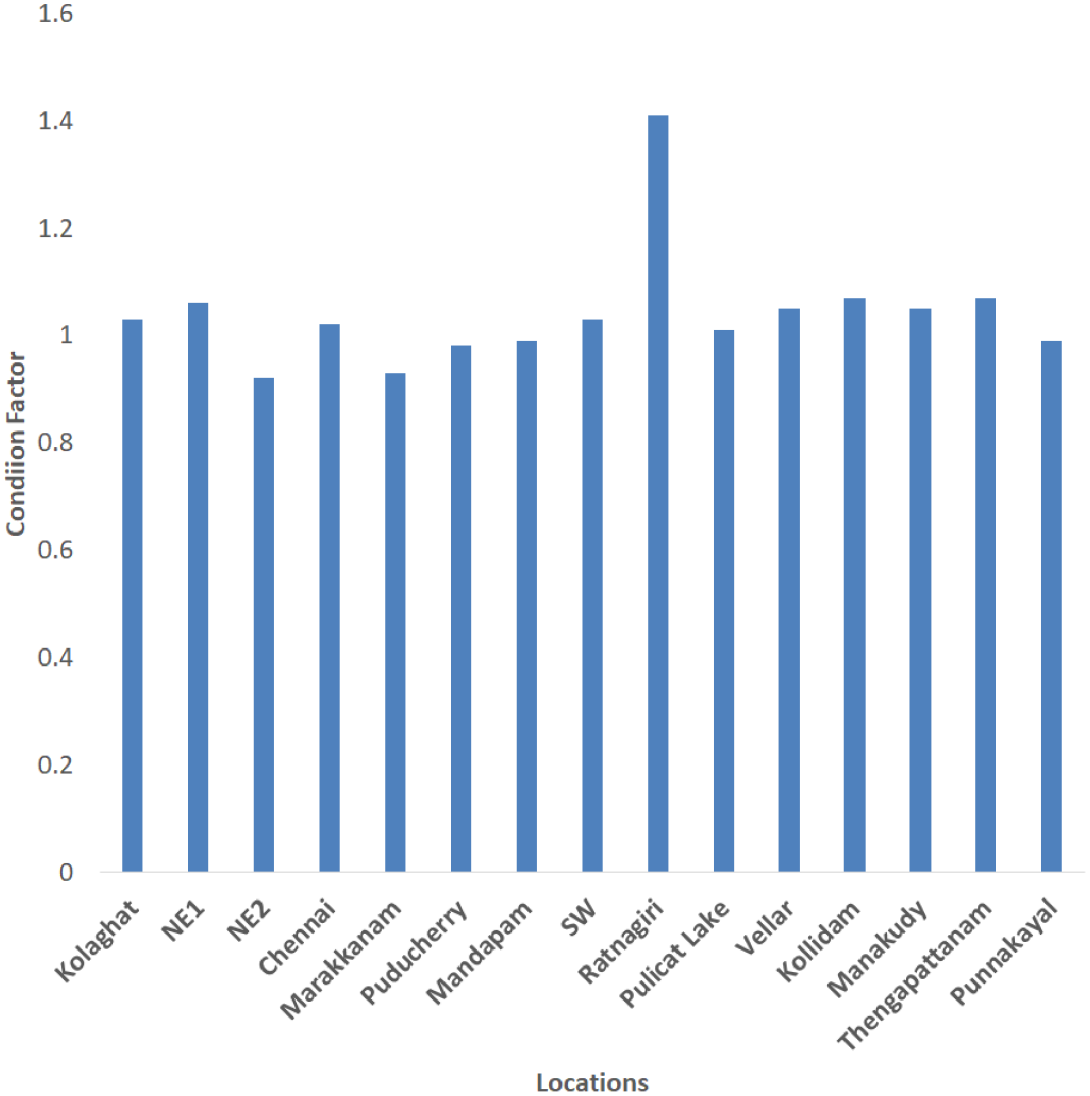
Location-wise depiction of condition factor (K) of *Mugil cephalus*.

**Figure 3:**
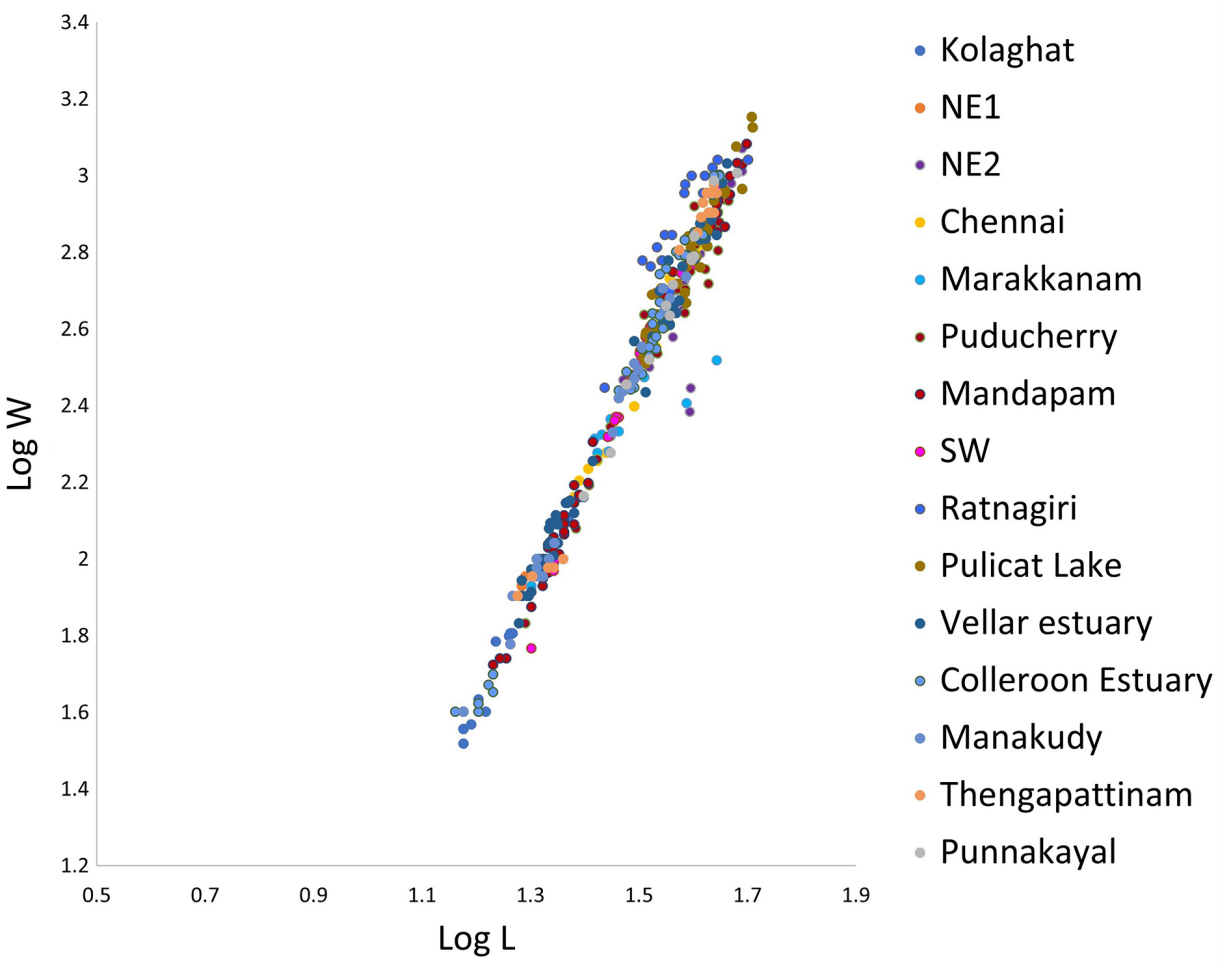
Linear regression of log weight on log length of *Mugil cephalus*.

Linear regression (LR) of the log-transformed weight on the length of the fish specimens provided the weight growth or mean values of estimates ‘b’ or ‘b’ (LR) for 15 locations. The ‘b’ values ranged from 2.235 (Ratnagiri) to 3.173 (Punnakayal) while the ‘a’ values ranged from 0.005321 (Punnakayal) to 0.22182 (Ratnagiri) (Table 2). The LWR parameters may vary if more samples are included. Positive allometric growth was observed in Chennai (3.109), Thengapattanam (3.121), and Punnakayal (3.173), while in SW (3.062) and Kollidam (3.069) an isometric growth pattern was observed. All other locations exhibited a negative allometric growth pattern. The coefficient of determination (R^2^) ranged from 0.80–0.99. The average value of Fulton’s condition factor K ranged from 0.92 (NE2) to 1.41 (Ratnagiri) (Fig. 2). A scatter plot of all observations across locations with Log L against Log W (Fig. 3) depicted a high correlation between the two variables or stronger relationship, as the data points were closer, forming a straight line when plotted.

**Table 2:** Parameters of the length-weight relationship and condition factor among different locations of India for *Mugil cephalus*.

Sl No.	Code	Regression parameters			Fulton’s condition factor (K)		Relative condition factor (K_n_)	
		a	b	R^2^	Range	Mean ± SD	Range	Mean ± SD
**FRESHWATER**								
1	Kolaghat	0.014632	2.87	0.97	0.89–1.19	1.03 ± 0.075	0.86–1.17	1.00 ± 0.071
**COASTAL**								
2	NE1	0.025398	2.72	0.96	0.86–1.26	1.06 ± 0.093	0.54–1.17	0.96 ± 0.152
3	NE2	0.017947	2.804	0.92	0.40–1.18	0.92 ± 0.169	0.46–1.23	1.02 ± 0.184
4	Chennai	0.006955	3.109	0.98	0.84–1.18	1.02 ± 0.083	0.83–1.15	1.00 ± 0.079
5	Marakkanam	0.018382	2.807	0.89	0.38–1.14	0.93 ± 0.155	0.44–1.25	1.02 ± 0.17
6	Puducherry	0.015642	2.863	0.97	0.68–1.31	0.98 ± 0.14	0.73–1.37	1.00 ± 0.141
7	Mandapam	0.010982	2.971	0.99	0.78–1.15	0.99 ± 0.078	0.79–1.16	1.00 ± 0.078
8	SW	0.008333	3.062	0.98	0.73–1.19	1.03 ± 0.091	0.73–1.18	1.00 ± 0.090
9	Ratnagiri	0.22182	2.235	0.80	0.87–1.81	1.41 ± 0.247	0.74–1.22	1.01 ± 0.151
**ESTUARY**								
10	Pulicat Lake	0.025948	2.740	0.92	0.78–1.31	1.01 ± 0.117	0.80–1.25	1.00 ± 0.110
11	Vellar	0.013086	2.934	0.99	0.79–1.31	1.05 ± 0.110	0.76–1.26	1.00 ± 0.104
12	Kollidam	0.008402	3.069	0.99	0.89–1.35	1.07 ± 0.116	0.83–1.29	1.00 ± 0.107
13	Manakudy	0.013782	2.915	0.99	0.94–1.26	1.05 ± 0.084	0.91–1.17	1.00 ± 0.076
14	Thengapattanam	0.006952	3.121	0.99	0.83–1.21	1.07 ± 0.114	0.82–1.19	1.00 ± 0.103
15	Punnakayal	0.005321	3.173	0.98	0.86–1.18	0.99 ± 0.087	0.88–1.15	1.00 ± 0.081

**Note:**

SD, Standard deviation.

### Multivariate analysis

#### Descriptive statistics of environmental variables

The mean, minimum, maximum, standard deviation (SD), and coefficient of variation (CV) computed for the gain in weight ‘b’ (LR) and nine environment variables (Table 3) reflected the variations in environmental parameters and weight change between the various locations.

**Table 3:** Estimate of ‘b’ or b (linear regression), coefficient of determination (R^2^) from regression, and distribution of climate variables (mean values for period 2002–17) over nine locations and four zones of India.

Zone	Location	b (LR)	R^2^	Climate variables								
				Sea surface temperature (°C)	Chlorophyll (mg/m^3^)	Salinity (ppt)	pH	DO (m mol/m^3^)	Nitrate (m mol/m^3^)	Silicate (m mol/m^3^)	Iron (m mol/m^3^)	Phosphate (m mol/m^3^)
N-E	Kakdwip	2.55	0.90	27.75	3.05	17.24	8.18	7.00	6.93	9.54	0.01	0.03
	Paradeep	2.89	0.96	27.89	2.67	27.79	8.10	6.85	8.27	7.97	0.00	0.04
S-E	Chennai	3.11	0.98	28.59	0.74	32.38	8.06	6.56	1.12	3.66	0.00	0.02
	Marakkanam	2.95	0.78	28.72	1.15	32.54	8.06	6.56	1.05	3.68	0.00	0.03
	Puducherry	2.89	0.96	28.80	1.59	32.59	8.06	6.57	1.04	3.65	0.00	0.04
	Cuddalore	3.19	0.99	28.77	1.25	32.61	8.06	6.56	1.00	3.61	0.00	0.03
	Mandapam	2.97	0.99	29.41	1.02	32.88	8.04	6.43	0.90	3.30	0.00	0.08
S-W	Karwar	3.21	0.98	28.49	2.36	34.97	8.04	6.51	0.34	2.61	0.00	0.08
N-W	Ratnagiri	2.24	0.79	28.29	3.48	35.14	8.05	6.42	0.08	2.97	0.00	0.07
	Mean	2.89		28.52	1.92	30.90	8.07	6.61	2.30	4.55	0.00	0.05
	SD	0.31		0.50	0.99	5.54	0.05	0.19	3.04	2.44	0.00	0.02
	CV	0.11		0.02	0.51	0.18	0.01	0.03	1.32	0.54	0.34	0.52
	Min	2.24	0.78	27.75	0.74	17.24	8.04	6.42	0.08	2.61	0.00	0.02
	Max	3.21	0.99	29.41	3.48	35.14	8.18	7.00	8.27	9.54	0.34	0.52

**Note:**

SD, Standard deviation; CV, Coefficient of variation; Min, Minimum; Max, Maximum.

The bivariate Pearson correlation coefficient of ‘b’ (LR) with nine environment variables was computed to observe the significance as a correlation coefficient value ≥0.57 and ≤−0.57 at *p* = 0.05 in a two-tailed distribution. The correlogram for SST with the other eight environment variables ranged from −0.80 to 0.65. All of the environmental variables studied had a significant correlation with SST, with the exception of phosphate, indicating that the environment data structure may have a multicollinearity problem (*i.e*., non-linearity, non-independence, non-normal). Hence, the PLS modelling was considered to be appropriate (Fig. S1, Table S1).

#### Determination of factors for the PLS model

PLS modelling determined seven factors by cross-validation between PRESS and RMSE. The model was based on three and four factors with PRESS residuals of 0.15 and −0.04, and relative changes in PRESS residuals of −0.32, and −1.27, respectively. Similarly, the RMSE results produced values for three and four factors as 1.46 and 1.70, respectively, and relative changes in RMSE as 0.06 and 0.16, respectively. The difference between cumulative variance (%) controlled by three and four factors for the explanatory variables data matrix was only 1.73%. It was evident that the model based on three factors had smaller values compared to that based on four factors and the cumulative variance of 1.73% was explained. Therefore, the three factor model (or PLS model) was observed to be suitable for the PLS analysis of covariations of explanatory (environmental) and response (weight growth) variables over nine locations (Table S2, Fig. S2).

#### Score scatterplot matrix of environment variables and fish weight growth of PLS model

##### Score scatterplot matrix of environment variables

**Figure 4:**
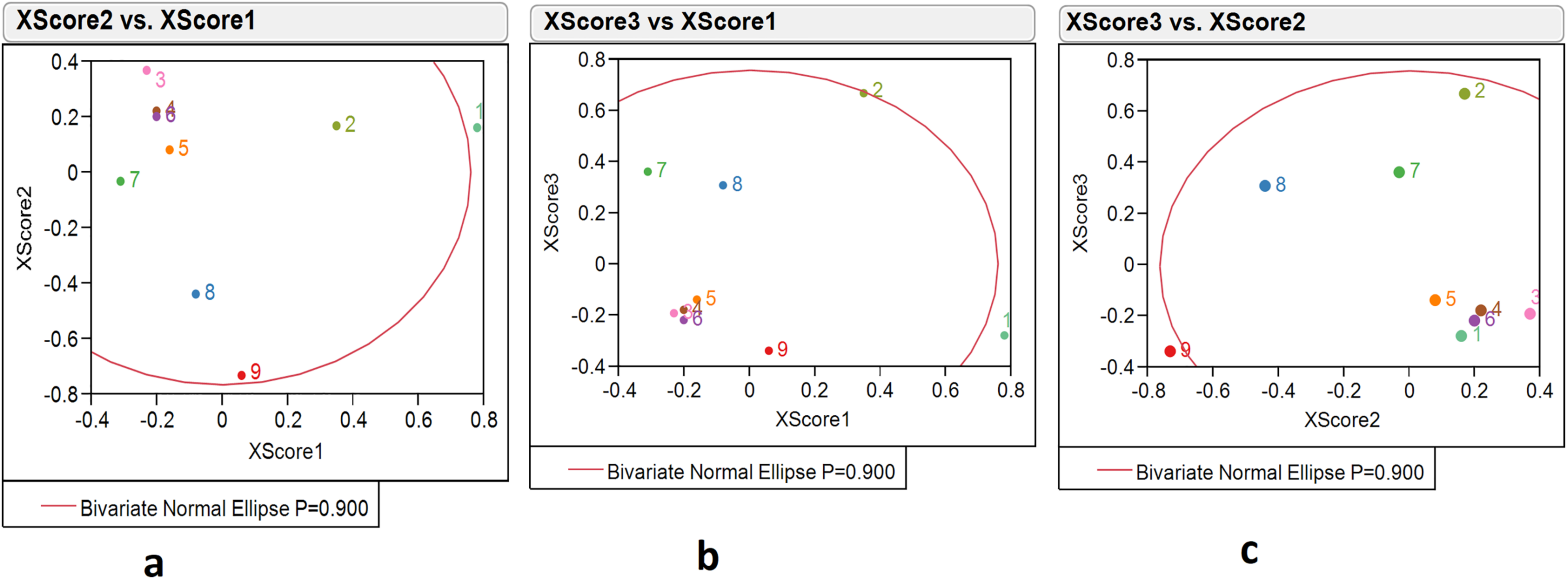
The 90% confidence ellipse distribution of nine locations with reference to climate variables scores (X scores 1, 2, 3) composing PLS factors (factors 1, 2, 3). 1, Kakdwip; 2, Paradeep; 3, Chennai; 4, Marakkanam; 5, Puducherry; 6, Cuddalore; 7, Mandapam; 8, Karwar; 9, Ratnagiri.

The PLS model with three factors illustrated the distribution of locations over environmental variable scores. An environment variable score composition was shown as X scores 1, 2, 3, while the distribution was represented by an ‘X score scatterplot matrix’. The score scatter plot matrix of environment variables indicated variations in nine environment variables over the nine locations. The X score 1 identified Kakdwip, Paradeep, and Mandapam with scores of 0.78, 0.35, and −0.31, respectively; X score 2 identified Chennai, Ratnagiri, and Karwar with scores of 0.31, −0.73 and −0.44, respectively; while X score 3 identified Paradeep, Mandapam, Karwar, and Ratnagiri with scores of 0.67, 0.36, 0.31, and −0.34, respectively (Table S3). The X score 1 *vs* 2 graph showed Kakdwip as an outlier and Ratnagiri on the boundary of the ellipse of the 90% confidence region. The graph from X score 1 *vs* 3 also indicated Kakdwip as an outlier but Paradeep on the boundary of ellipse 90% confidence region. X score 2 *vs* 3 indicated Ratnagiri as the outlier. Thus, through score analysis and the ‘X Score scatterplot matrix’, we determined that Kakdwip, Paradeep, and Ratnagiri had higher variations in environment variables as compared to other locations (Table S3, Figs. 4A–4C).

##### Score scatterplot matrix for fish weight growth

**Figure 5:**
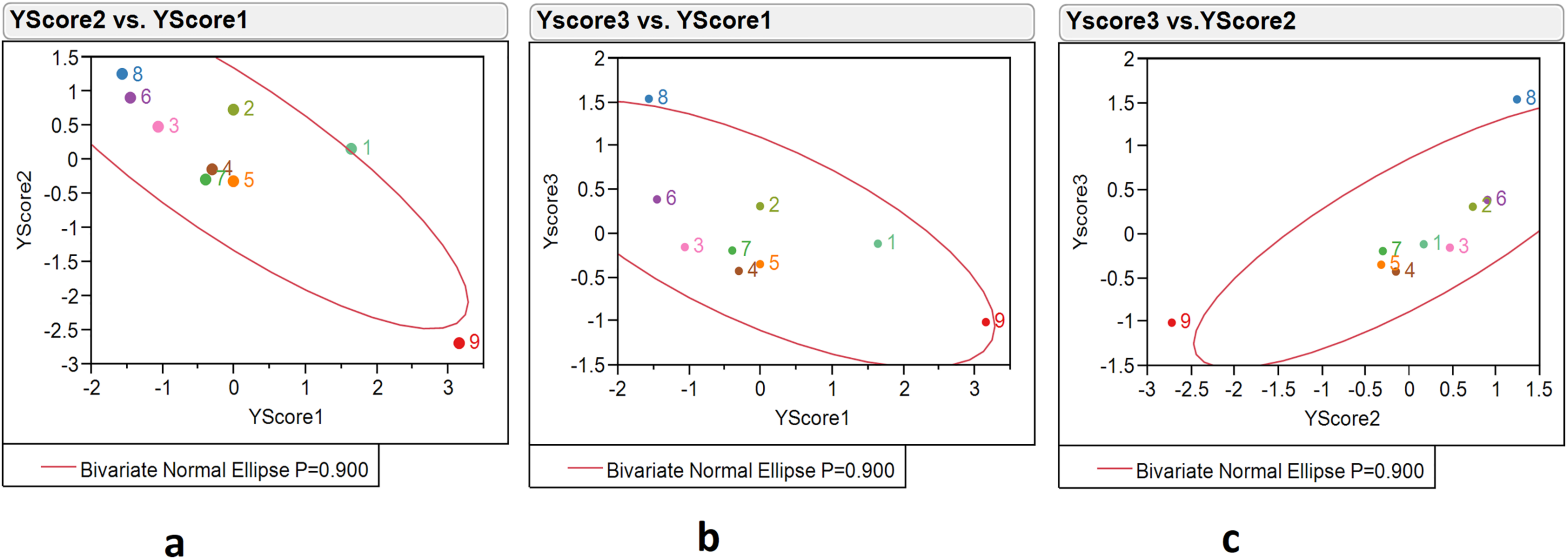
The 90% confidence ellipse distribution of nine locations with reference to fish weight growth or response variable scores (Y scores 1, 2, 3) composing PLS factors (factors 1, 2, 3). 1, Kakdwip; 2, Paradeep; 3, Chennai; 4, Marakkanam; 5, Puducherry; 6, Cuddalore; 7, Mandapam; 8, Karwar; 9, Ratnagiri.

The PLS model with three factors was used to illustrate the distribution of fish weight grown (or response) variable scores over location. The response variable score composition was shown as Y scores 1, 2, 3, and the ‘Y score scatter plot matrix’ was used. The Y score 1 identified Ratnagiri, Kakdwip, and Karwar as areas with the highest scores of 3.15, 1.65, and −1.56, respectively and the Y score 2 identified Karwar and Ratnagiri with scores of 1.24 and −2.71, respectively. The Y score 3 identified Karwar and Ratnagiri with scores of 1.54 and −1.01. The Y score 1 *vs* 2 graph identified Ratnagiri as an outlier, Kakdwip on the boundary, and other locations within the boundary of the ellipse 90% confidence region. Both the Y score 1 *vs* 3 and the Y score 2 *vs* 3 identified Ratnagiri and Karwar as outliers. All other locations were within the 90% confidence region of the ellipse. Thus, Karwar and Ratnagiri were outliers with better fish weight growth, followed by Kakdwip and Paradeep (Table S3, Figs. 5A–5C).

#### Distance plot model for environment variables and weight growth of PLS model

**Figure 6:**
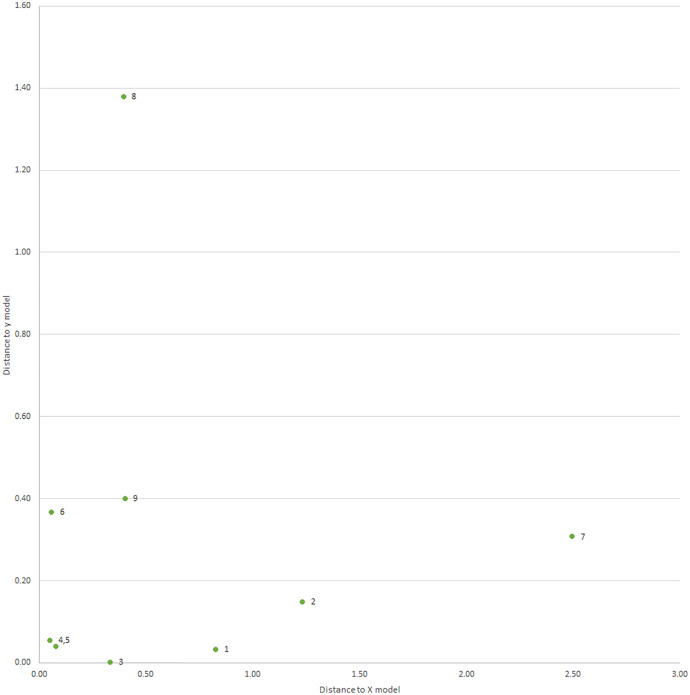
Distance plot distribution of nine locations with reference to distance to X model *vs* distance to Y model. 1, Kakdwip; 2, Paradeep; 3, Chennai; 4, Marakkanam; 5, Puducherry; 6, Cuddalore; 7, Mandapam; 8, Karwar; 9, Ratnagiri.

The PLS model with a distance plot analysis of the environment (X) model showed that the greatest distances for Mandapam (2.50), and Paradeep (1.23), and the shortest distance for Marakkanam (0.05) (Table S3). Similarly, the distance plot analysis of the weight growth (Y) model identified the greatest distance for Karwar (1.38), followed by Ratnagiri (0.40) (Table S3). The seven remaining locations were located differently, with the exception of Marakkanam and Puducherry, which were close due to similar distances from the Y model. Karwar and Ratnagiri had the greatest distance from the Y model with lower distances from the X model, revealing lower changes in non-favorable environmental variables for better weight growth and greater changes for favorable variables. (Table S3, Fig. 6).

#### Outlier analysis of fish weight growth linked to environmental variations

According to Hotelling T^2^ analysis with an upper control line of 5.44, Kakdwip was an outlier with a T^2^ value of 5.71 (Table S3, Fig. S4). The outlier analysis identified Mandapam, Karwar, and Ratnagiri as ideal locations for fish growth with favorable environmental variables considering the environmental variable score analysis, response variable score analysis, model distance, and optimum T^2^ value (Figs. S3A and S3B).

#### Factors for environmental variables and fish weight growth of PLS model

##### Factors for total variations of environmental variables and fish weight growth

**Figure 7:**
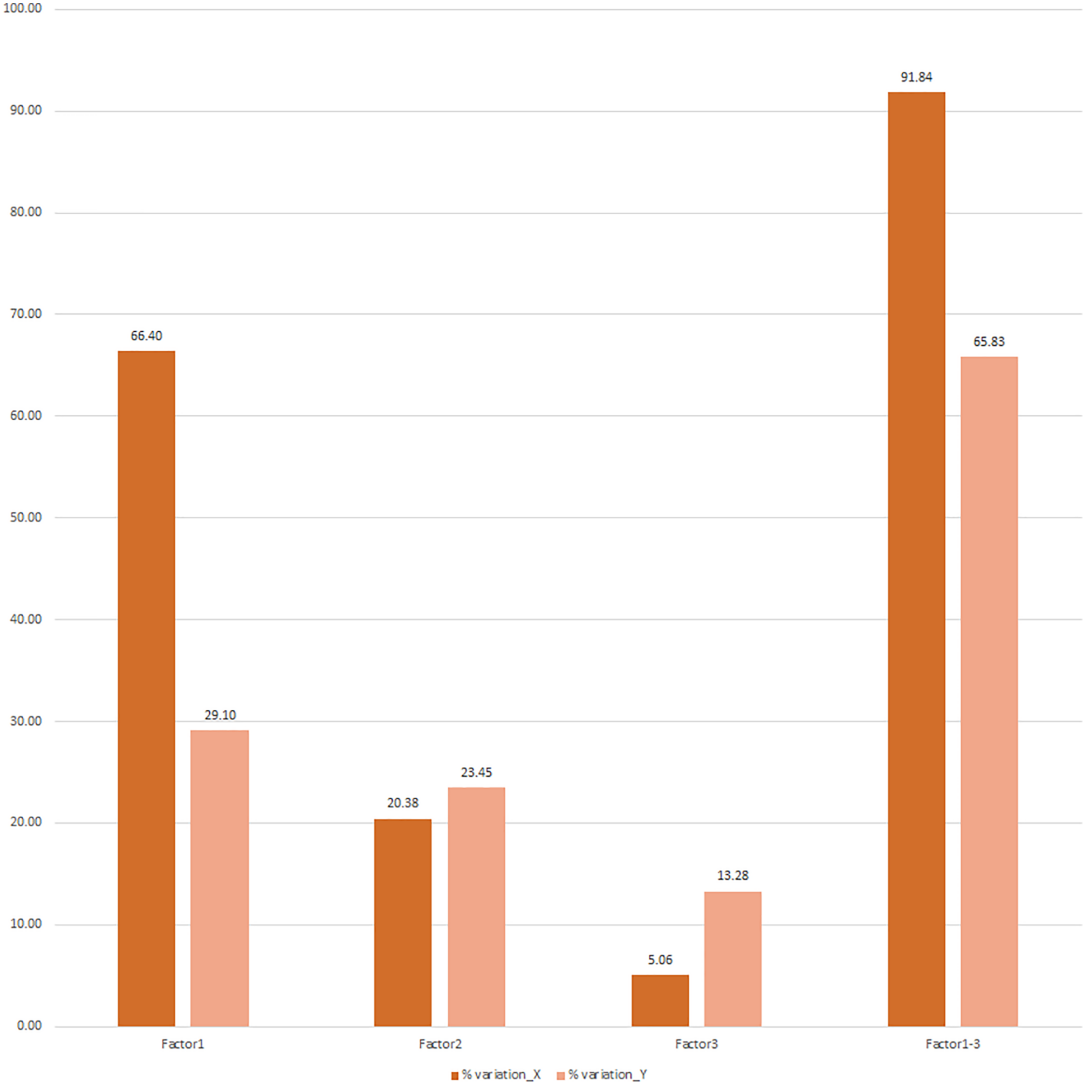
Percent variation explained in X (environment variables) and Y response (weight growth) through three PLS factors (factors 1, 2, 3) derived from PLS analysis of response variable matrix & explanatory variables matrix.

The three identified PLS factors, 1, 2, and 3 explained the variations in environmental variables (X effect) as 66.40%, 20.38%, and 5.06%, respectively. These were 91.84% of the 9 × 9 data matrix, cumulatively. Similarly, PLS factors 1, 2, and 3 explained the variations in weight growth variable (Y effect) as 29.10%, 23.45%, and 13.28%, respectively, with a cumulative variation of 65.83% on the weight growth for the 9 × 1 data matrix (Fig. 7).

##### Factors for variations in environmental variables

The percentage (%) variation of the PLS factors (1, 2, 3) for nine environmental variables and the total variation is shown in Table 4 (Fig. S5). PLS factors 1, 2, and 3 controlled 0.79–0.99% of the variation in all environmental variables over the nine locations.

**Table 4:** Factor compositions: factors 1, 2, and 3 loadings (X loadings 1, 2, 3), weights (X weights 1, 2, 3), coefficients and variable of importance (value > 0.80) of climate variables from PLS modelling of response variable (weight growth-Y) with explanatory variables.

Factors compositions and contribution	Climate variables (X)								
	Sea surface temperature (°C)	Chlorophyll (mg/m^3^)	Salinity (ppt)	pH	DO (m mol/m^3^)	Nitrate (m mol/m^3^)	Silicate (m mol/m^3^)	Iron (m mol/m^3^)	Phosphate (m mol/m^3^)
Factor 1	78.76	56.53	76.85	84.92	77.24	68.67	81.92	68.81	3.88
Factor 2	0.22	39.33	15.80	11.54	20.48	13.64	14.40	2.85	65.13
Factor 3	0.09	0.01	0.49	1.82	0.98	14.04	2.18	8.66	17.27
Factors (1,2,3)	79.07	95.87	93.15	98.28	98.69	96.35	98.51	80.32	86.28
X loadings 1	−2.51	2.13	−2.48	2.61	2.49	2.34	2.56	2.35	−0.56
X loadings 2	0.13	−1.77	−1.12	0.96	1.28	1.04	1.07	0.48	−2.28
X loadings 3	0.09	0.03	0.20	−0.38	0.28	1.06	0.42	−0.83	1.18
X weights 1	−0.79	0.67	−0.78	0.82	0.78	0.74	0.80	0.74	−0.18
X weights 2	0.04	−0.56	−0.35	0.30	0.40	0.33	0.34	0.15	−0.72
X weights 3	0.03	0.01	0.06	−0.12	0.09	0.33	0.13	−0.26	0.37
Coefficients for response variable (Y)	0.05	−0.78	0.09	−0.24	0.47	0.32	−0.02	−0.29	0.20
VIP-variable of importance	0.99	2.3	0.66	0.84	1.2	0.93	0.66	0.91	1.34

##### Environmental variables loadings analysis over PLS factors

**Figure 8:**
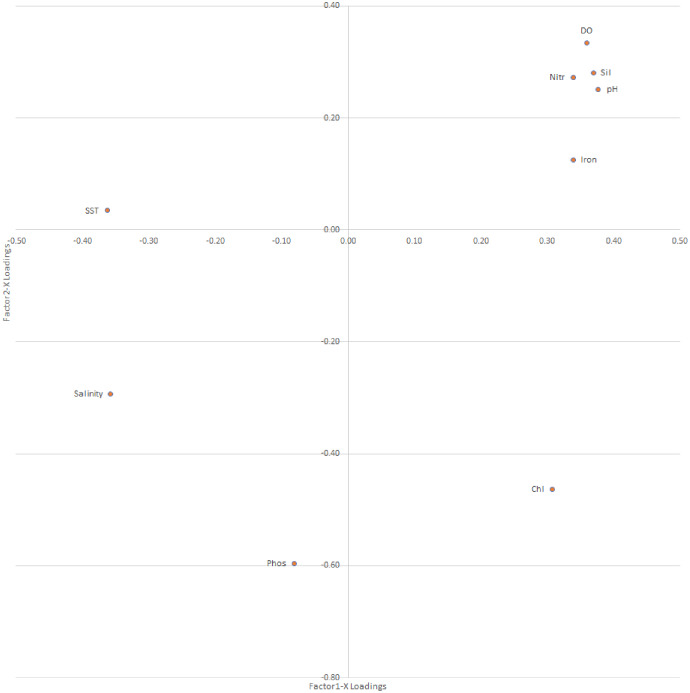
Loading distribution plot for environment variables over PLS-factors; factor 1 *vs* factor 2, from PLS analysis of response variables (weight growth) on explanatory variables (climate variables). SST, sea surface temperature; Sil, silicate; Nitr, nitrate; Phos, phosphate; DO, dissolved oxygen.

The bivariate distribution of the loadings over PLS factor 1 *vs* 2 *vs* 3 explains the role of the environmental variables (loadings) over the PLS factors, which can be used to explain the variations in environmental variables affecting fish weight growth in different locations (Table 4, Fig. 8).

##### Environmental variable weight analysis over PLS factors

The bivariate distribution of X weightage over PLS factor 1 *vs* factor 2 explains the distinct role of the environmental variable (weightage) in fish weight growth in different locations (Table 4, Fig. S6).

##### Environmental variables contribution (coefficients) in weight growth

The contribution of environmental variables in estimating the response variable (Y) showed that five variables, including SST, salinity, DO, nitrate, and phosphate, had positive coefficients. In contrast, chlorophyll, pH, silicate, and iron had negative coefficients to explain the favorable conditions for fish weight growth in different locations (Table 4, Fig. S7).

##### Distribution of the environmental variable in the variable of importance plot (VIP)

**Figure 9:**
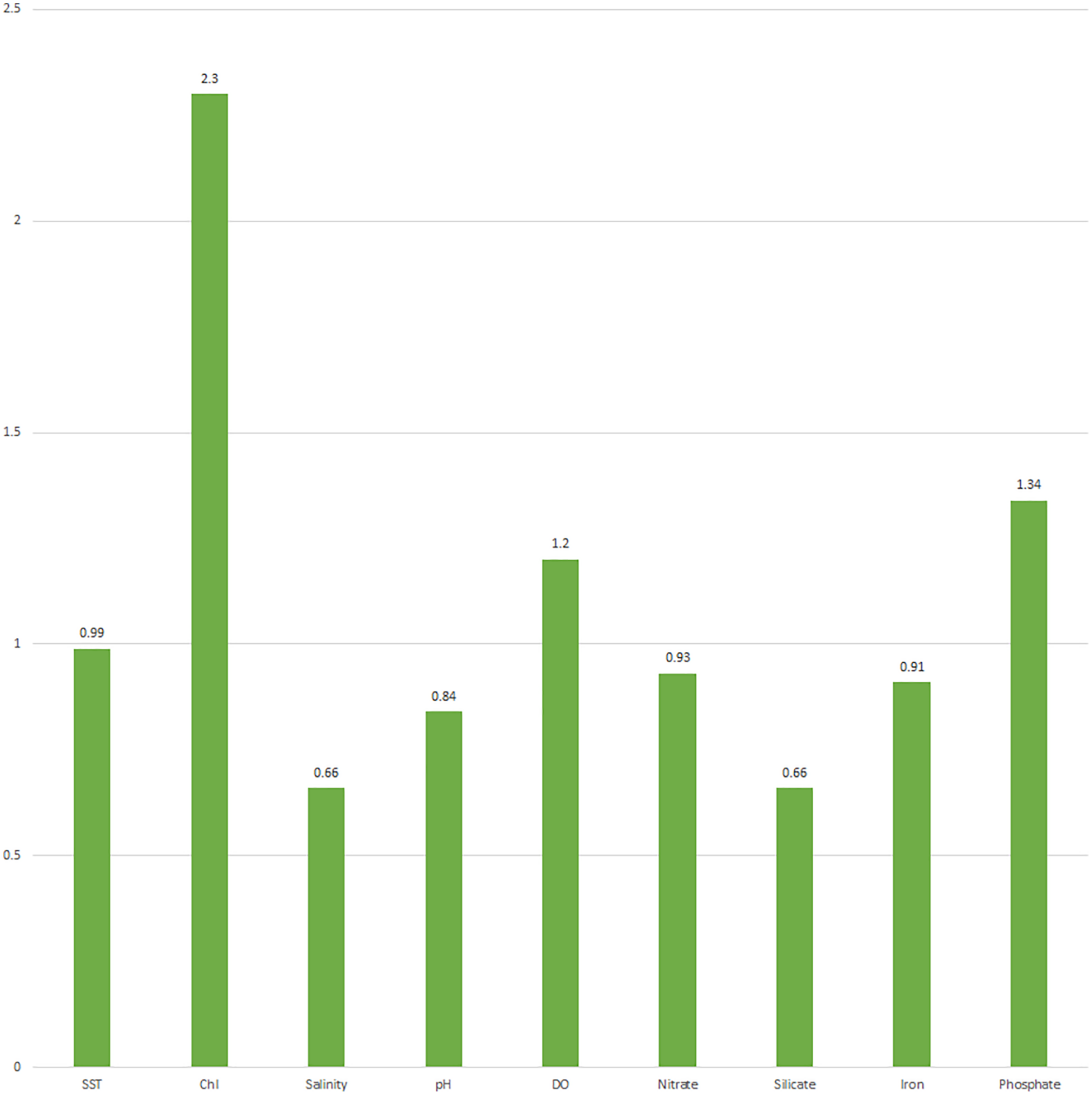
Variable importance plot (VIP) of environment variables derived from PLS analysis of response variable (weight growth) on explanatory variables (nine climate variables). SST, sea surface temperature; Chl, chlorophyll; DO, dissolved oxygen.

The coefficients for environmental variables greater or equal to 0.80 were called “variables of importance for estimating fish weight growth”. Five of the nine environmental variables (SST, chlorophyll, DO, nitrate, and phosphate) contributed as coefficients (≥0.80) and were denoted as a “variable of importance” for estimating weight growth between the nine locations. A positive coefficient with a higher value and a negative coefficient with a lower value had favorable impacts on fish weight growth between locations (Table 4, Fig. 9).

#### Prediction of weight growth by PLS model

PLS modelling for weight growth on environmental variables generated a covariance matrix and three PLS factors. To predict the weight growth of fish specimens over nine locations, we used variations that were visualized through scores, loadings, weights, coefficients, and the variable of importance for nine environmental variables over three factors. The PLS model developed for the prediction of weight growth-b (PLS) is as follows:

$\eqalign {&`\rm {b}' \rm(PLS)=10.86+0.3140*\{0.093*SST-0.784*Chl+0.015*Salinity-5.39* \cr& pH+2.41*DO+0.105*Nitrate-0.007*Silicate-86.38*Iron+8.60*Phosphate\}}$

The PLS model was used to predict ‘b’ (PLS), which had values of PRESS 0.15, and RMSE −1.46 and explained the cumulative variance of environmental variables for approximately 91.84% and the variance in fish weight growth for 65.83% (Table S2). This PLS model used a distribution pattern of environmental variables over scores, coefficients, weightages, and loadings. The model reflected favorable environmental variables with positive coefficients such as SST, salinity, DO, nitrate, and phosphate. However, we identified less favorable negative coefficients (chlorophyll, pH, silicate, and iron), which can be managed for higher growth in fish weight.

**Figure 10:**
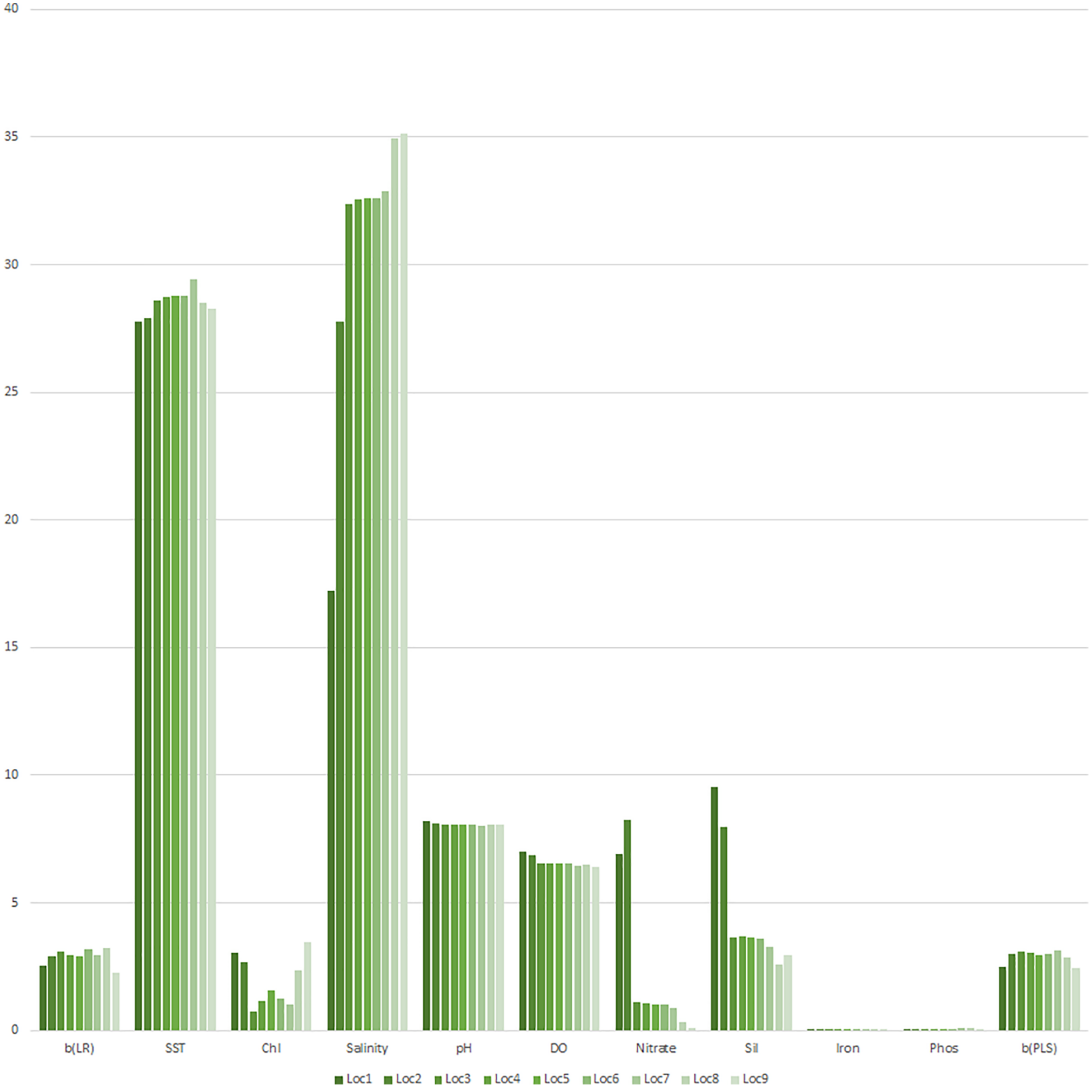
Distribution of b(LR) and environment variables along with b(PLS). SST, sea surface temperature; Chl, chlorophyll; Sil, silicate; Phos, phosphate; DO, dissolved oxygen; 1, Kakdwip; 2, Paradeep; 3, Chennai; 4, Marakkanam; 5, Puducherry; 6, Cuddalore; 7, Mandapam; 8, Karwar; 9, Ratnagiri.

**Figure 11:**
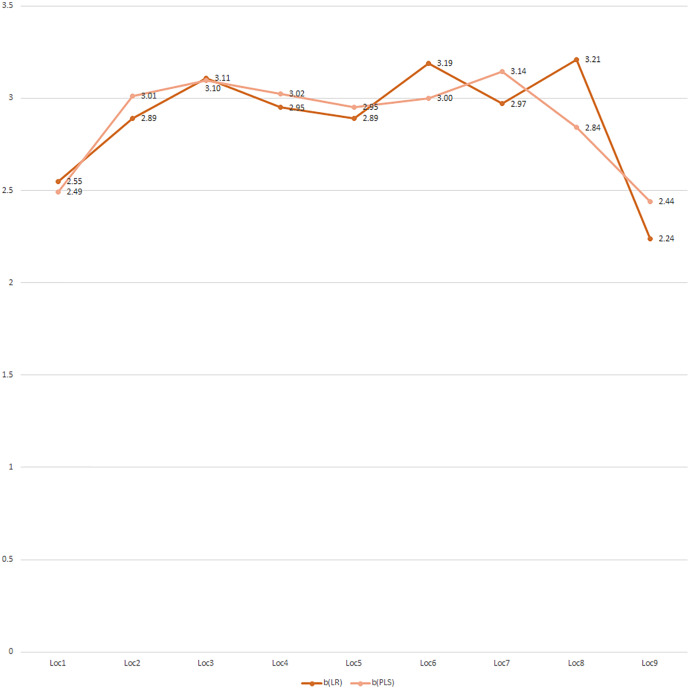
Correspondence between b(LR) and b(PLS) through the partial least squares (PLS) model. 1, Kakdwip; 2, Paradeep; 3, Chennai; 4, Marakkanam; 5, Puducherry; 6, Cuddalore; 7, Mandapam; 8, Karwar; 9, Ratnagiri.

The graphical presentation of ‘b’ (LR), the nine environmental variables, and ‘b’ (PLS) informed environmental variables influencing weight growth ‘b’ (PLS) varied over locations (Fig. 10). Further, the distribution of weight growth ‘b’ (LR) and ‘b’ (PLS) had noticeable differences for Mandapam (seven), Karwar (eight), and Ratnagiri (nine), explaining the presumed role of environmental variations in influencing weight growth for these locations (Fig. 11).

## Discussion

### Length-weight regression analysis

The present study aimed to identify the environmental factors that influence the growth and well-being of *M. cephalus* in Indian waters. LWR correlates the mathematical relationship between the variables, length, and weight of the fish (Lawson, 2011; Thulasitha & Sivashanthini, 2012). The value of ‘b’, which is the regression coefficient, is typically 3.0 for an ideal fish (Martin, 1949). The larger the distance from 3.0, the greater the change in form or condition (Hile, 1936). The variation in the ‘b’ value for a species may be due to a single effect or the synergistic effects of many factors such as body health, habitat, extent of stomach fullness, sex, gonadal maturity stage, specimen number, collection time, or differences in the observed length range (Froese, 2006). LWR is an essential tool that provides insight into growth patterns (Ighwela, Ahmed & Abol Munafi, 2011). The regression coefficient ‘b’ was taken as an index of growth for *M. cephalus* and compared it with the environmental factors collected for various coastal locations across India. LWR is influenced by body size, range, sample size, and seasonal abundance and the detailed analysis of our study provides important information for the conservation management of this resource. An attempt to derive a relationship between fish growth and environmental factors, and to identify positive impacting variables, may provide information about the performance of this vital candidate species for mariculture. We created a model to best describe the interaction between growth and the corresponding environment variables of *M. cephalus*.

The ‘b’ value was found to be consistent with the expected range of 2.5 to 3.5 (Froese, 2006) and was significant (*p* ≤ 0.01). The regression coefficient was highest for Chennai (3.109) among the coastal locations studied. Chennai is a key location for marine fish landings and many researchers have analyzed the LWR of fish found in this location (Martin, Kuppan & Kalaichelvi, 2016; Das & Bhaskar, 2020; Kodeeswaran et al., 2020). Vaitheeswaran, Malathi & Felix (2016) reported the regression coefficient to be 1.0368 from Chennai, while in the present study, it was observed to be 3.109. Better growth was also observed in the SW location (3.062) inclusive of Karwar and Goa. The southwest monsoon and upwelling provide enormous amounts of food for fish (Jadhav & Rathod, 2014) and Karwar is subjected to great fluctuation. There is also a cluster of islands in Karwar Bay, making the location ideal for fish growth. Reports of the dominance of mackerel fishery on the Karwar coast (Pradhan, 1956) have been widely studied. The presence of mudflats and mangroves, tropical conditions characterized by high temperature, extended photoperiod, and long flushing periods, are conducive to greater biological productivity in Goa (Sreekanth, Lekshmi & Singh, 2015). In the present study, the favorable habitat and environmental conditions in Karwar and Goa are reflected in better growth performance in the SW region. All the estuarine locations also displayed better growth with a ‘b’ value ranging from 2.740 to 3.121. The topography and fertility of estuaries make them the most ideal sites for fish (Hickling, 1971) as there is an abundant source of food, enabling better growth and thus a higher regression coefficient. Rangaswamy (1973) and Murugan et al. (2012) studied the *M. cephalus* population from Pulicat Lake and the Vellar estuary, respectively and found almost similar values to that of the present study. The negative allometric results of Kolaghat (2.87) in the current investigation are comparable to findings by Hora & Pillai (1962), who reported a ‘b’ value of 2.8779 from the Hooghly–Matlah estuary. Rao & Ramesh (2013) reported negative allometry for grey mullet sampled from the east coast of Andhra Pradesh (2.81).

The LWR of *M. cephalus*, a cosmopolitan species, has been studied by many researchers worldwide. Dankwa (2011) studied the LWR of the grey mullet and reported the ‘b’ value to be 3.1387 from the Volta estuary and 3.1708 from the Pra estuary in Ghana. Khayyami et al. (2014) reported the ‘b’ value of the grey mullet from Bandar Abbas Port and Qeshm Island to be 2.9118 and 2.9018, respectively. The grey mullet from El-Ghazala Lagoon, Libya was characterized as 2.892 (Mohammed et al., 2016) and the condition factor was highest during winter (1.129) and lowest during autumn (0.987). The regression coefficient value of the grey mullet has marginally negative allometry. Ao, Omolara & Eteobong (2017) reported the ‘b’ value of the grey mullet to be 2.7745 from Lagos Lagoon, Nigeria. Awan et al. (2017) documented the ‘b’ value of *M. cephalus* from Narreri Lagoon, Pakistan, to be 2.931 and Zubia et al. (2014) reported a value of 2.669 from the Karachi coast. Reis & Ateş (2019) studied the LWR from Köyceğiz Lagoon in Turkey and reported the ‘b’ value to be 2.95 and the condition factor to range from 0.66 to1.22.

### Fish growth and environment variables

Synergistic interactions between the growth potential and environmental conditions are important for recruitment success (Rountrey et al., 2014). Fish typically respond to environmental change through alterations in their growth (Rountrey et al., 2014). In the present study, the PLS method was employed to predict the response of fish weight growth (‘b’) to environmental variables and identify patterns of interaction between variables affecting fish growth. Interestingly, six environment variables including SST, salinity, DO, nitrate, silicate, and phosphate positively impacted *M. cephalus*. Earlier studies also reported the positive impact of temperature on somatic growth (Brander, 1995; Heather et al., 2018; Gamperl et al., 2019; Huangab et al., 2021). This may be due to the increased production of benthos and plankton (Thackeray et al., 2013; Tao et al., 2015), which indirectly influenced the energy intake and growth of the studied fish (Pörtner & Farrell, 2008; Prokešová et al., 2020). The pH, DO, and silicate (Viadero, 2005; Bajaj, 2017; Zhang et al., 2017) favors the production of diatoms and algal matter in water, which forms the primary food of *M. cephalus* (Lavanya et al., 2018). Similarly, a large chain-forming diatom is also associated with regions with high nitrate (Olofsson et al., 2019) and phosphate (Dell’Aquila et al., 2020) content, leading to higher fish production. Additionally, the ideal environmental characteristics documented in the present study corresponded with the favorable conditions reported by Kumar et al. (2021) for the nursery rearing of *M. cephalus* for mariculture. We found that the environmental variables, chlorophyll, and iron were observed to have a negative role in the growth of *M. cephalus*. Increased concentrations of iron and organic carbon often cause water browning and decreased light penetration (Creed et al., 2018), which may result in reduced fish growth and production (van Dorst et al., 2019). Similarly, an increased chlorophyll content impacts light availability, affecting the primary production and altering the secondary consumer communities (zoobenthic and zooplankton invertebrates), which also impacts fish growth (van Dorst et al., 2020). However, it is pertinent to mention that this study considered the environment variables individually, and the synergistic interaction between them was not considered.

### Location effect on fish growth due to environmental variation

Based on environment variables, location-linked environment score analysis, and response (fish growth) score analysis, deviation from model, and outlier analysis, the present study identified Mandapam, Karwar, and Ratnagiri as having significantly higher fitness for mariculture of *M. cephalus* to their environment when compared to the other six locations. Mandapam coast is considered one of India’s rich biodiversity zones as it is located within the Gulf of Mannar Biosphere (Gopakumar, Sulochanan & Venkatesan, 2009). Many studies on plankton (Deepika et al., 2019), seaweeds (Mantri, Kavale & Kazi, 2020), coral (Ramesh et al., 2020), *etc*., have been conducted in this immensely diverse area. The coast of Mandapam has a relatively shallow depth (0.5–3.0 m) (Edward et al., 2008), enabling greater light penetration and, thus, greater productivity. Productivity variations alter plankton growth (Fox et al., 2020; Chandran et al., 2021) and play a positive role in the growth and well-being of fish. The lagoon at Mandapam is the primary source of mullets in the area (Luther, 1963). It should also be noted that there are no significant river inflows on the Mandapam coast and it is designated as a beach environment with less influence of rivers (Angusamy & Rajamanickam, 2006). Additionally, the presence of islands also endorses low energy conditions leading to shoal formation (Angusamy & Rajamanickam, 2006). The absence of significant river inflow also favors the locations of the Western coast, Karwar and Ratnagiri, making them the optimum locations for the culture initiation of *M. cephalus*. It is also noteworthy that cage culture experimental trials of mullet carried out at various locations across India by ICAR-Central Marine Fisheries Research Institute also identified Karwar as having more favorable environmental conditions and water quality (Philipose et al., 2012). Ratnagiri, with its ample fishery resources (Kumar et al., 2011; Pakhmode et al., 2013; Prabhu, Zodape & Sharma, 2021) and favorable environment variables had the highest condition factor in the present study, which is helpful for the mariculture of *M. cephalus*. River inflows create highly energetic and dynamic sedimentary environments with excellent spatial and temporal gradients in physical parameters like salinity and suspended sediment concentration (Dyer, 1997). Increased sediment concentration eventually leads to increased turbidity and conductivity, reducing the habitat suitability for fish occurrence and growth (Chandran et al., 2019). This could be why Kakdwip was recommended as an outlier location in the present study. Kakdwip is highly influenced by the Hooghly-Matlah estuary of the Ganga-Bhagirathi River system leading to huge variations in salinity (Roy & Nandi, 2012) and sedimentation (Tuhin, Gupinath & Sugata, 2003) that makes it relatively unsuitable for fish. Barman et al. (2005) has also studied the effects of salinity on the growth of *M. cephalus*.

### Model formulation

A PLS model associating weight growth (response variable) and environment variables (explanatory variables) was created and used to predict the weight growth over location by associating the ‘b’ matrix with environment variables. The model developed in the present study has advantages over the linear regression model, as it does not require linearity, homoscedasticity, independence, and normality in the data structure. The PLS model can address the issues of multicollinearity (non-linearity) and any constraints on the explanatory and response variables (Krugmann et al., 2020). PLS modelling, in the present study, generated factors (PLS factors) from the covariance of the data matrix of the response variables and the data matrix of the explanatory variables, which could help in understanding the relationship between the two in a covariance data structure (Höskuldsson, 1998; Falk & Miller, 1992; Hair et al., 2014). The PLS model reflects the distribution pattern of explanatory variables through scores, coefficients, weightages, and loadings.

This model may be used to predict the response variable (fish growth) with explanatory (environment) variables in different locations. The increase in fish weight may be obtained by managing the positive or negative impacts of environmental variables in different locations. This method has applicability in multi-collinear data, which is a common problem. Any number of equal or unequal observations for explanatory or response variables may be used. This model employs a covariance matrix of both data matrices (explanatory and response variables) that generates latent factors or PLS factors. The selection of a suitable number of factors (PLS factors), variations in explanatory variables (environment variables) and response variables (weight growth), and the association between them can be explained based on scores, loadings, weightage, coefficients, and variable of importance.

## Conclusion

The length-weight analysis of *M. cephalus* displayed an allometric growth pattern in wild. All of the estuarine and coastal locations, Mandapam, and the SW zone showed higher ‘b’ values, indicating better growth. The condition factor also revealed the health conditions of this species in Indian waters in relation to physical and biological conditions, growth, and other biological parameters. Environmental parameters, namely SST, salinity, DO, nitrate, silicate, and phosphate, positively impacted the growth of *M. cephalus*. The PLS model created in the present study provides an association of weight growth and environment variables, can predict fish growth with the environmental parameters, and resolve the multicollinearity of data prevalent in nature. Such baseline information for the biological, population, and aquaculture studies of *M. cephalus* will help develop science-based strategies and policy decisions for the conservation and management of wild populations.

## Supplemental Information

10.7717/peerj.14884/supp-1 Supplemental Information 1 Pearson correlation coefficient for weight growth b(LR) and climate variables. Tested for significance, as correlation coefficient (>0.57, <−0.57, *p* = 0.05, 2-tailed)
Correlation of SST with other variables observed significant, represented as bold

10.7717/peerj.14884/supp-2 Supplemental Information 2 Determination of number of factors by Root mean square error (RMSE) & Predicted residual error sum of squares (PRESS) from PLS modelling of response variable and explanatory variables.

10.7717/peerj.14884/supp-3 Supplemental Information 3 The distribution of climate variable scores (X Scores 1,2,3,) and response variable scores (Y Scores1, 2, 3), distance from model X, Y, predicted weight growth- b(PLS) by 3-factors PLS model, derived from partial least squares (PLS) analysis of weight gro.

10.7717/peerj.14884/supp-4 Supplemental Information 4 Correlogram for climate variables, bivariate correlation coefficient of Sea Surface Temperature with other environment variables. Chl: Chlorophyll, Sil: Silicate, Phos: Phosphate, DO: Dissolved Oxygen

10.7717/peerj.14884/supp-5 Supplemental Information 5 Distribution of relative change in root mean square error (RMSE), predicted residual error sum of squares (PRESS) over factors in cross-validation.

10.7717/peerj.14884/supp-6 Supplemental Information 6 Distance plot distribution of locations with reference to (a) distance to X model (environment model) (b) distance to Y model (weight growth model). 1. Kakdwip 2. Paradeep 3. Chennai 4. Marakkanam 5. Puducherry 6. Cuddalore 7. Mandapam 8. Karwar 9. Ratnagiri

10.7717/peerj.14884/supp-7 Supplemental Information 7 Hoteling T^2^ test for outlier analysis for distribution of nine locations. 1. Kakdwip 2. Paradeep 3. Chennai 4. Marakkanam 5. Puducherry 6. Cuddalore 7. Mandapam 8. Karwar 9. Ratnagiri

10.7717/peerj.14884/supp-8 Supplemental Information 8 Histogram on percent variation explained for climate variables through PLS-factors (Factors 1,2,3). SST: Sea Surface Temperature, Chl: Chlorophyll, DO: Dissolved Oxygen

10.7717/peerj.14884/supp-9 Supplemental Information 9 Loadings distribution plot for environment variables over PLS-factors; (a) Factor 1 *vs* Factor 3 (b) Factor 2 *vs* Factor 3 from PLS analysis. SST: Sea Surface Temperature, Chl: Chlorophyll, DO: Dissolved Oxygen

10.7717/peerj.14884/supp-10 Supplemental Information 10 Distribution of weightage of environment variables over Factor 1 *vs* Factor 2 from PLS analysis. SST: Sea Surface Temperature, DO; Dissolved Oxygen

10.7717/peerj.14884/supp-11 Supplemental Information 11 Coefficients distribution of environment variables in estimation of response variable (weight growth), as coefficients estimated from PLS analysis. SST: Sea Surface Temeperature, Chl: Chlorophyll, DO: Dissolved Oxygen

10.7717/peerj.14884/supp-12 Supplemental Information 12 Raw data. Each location wise log converted length and weight data of the samples collected during the study.

## Additional Information and Declarations

### Field Study Permissions

The following information was supplied relating to field study approvals (*i.e*., approving body and any reference numbers): ICAR-National Bureau Of Fish Genetic Resources, Lucknow

### Data Availability

The following information was supplied regarding data availability: The raw data is available in the Supplemental File.
