# Supplementary material for: Evaluating the influence of environmental variables on the length-weight relationship and prediction modelling in flathead grey mullet, Mugil cephalus Linnaeus, 1758
Source: PeerJ. 2023 Feb 24;11:e14884. doi: 10.7717/peerj.14884 (PMC9969857; doi:10.7717/peerj.14884)
Supplement: Supplemental Information 1 — Tested for significance, as correlation coefficient (>0.57, <−0.57, p = 0.05, 2-tailed) Correlation of SST with other variables observed significant, represented as bold [file peerj-11-14884-s001.docx]

**Supplemental Table 1.** Pearson correlation coefficient for weight growth b(LR) and climate variables

|  | b(LR) | Sea Surface Temperature | Chlorophyll | Salinity | pH | DO | Nitrate | Silicate | Iron | Phosphate |
| --- | --- | --- | --- | --- | --- | --- | --- | --- | --- | --- |
| b(LR) | 1.00 | 0.46 | -0.74 | 0.31 | -0.38 | -0.17 | -0.21 | -0.31 | -0.40 | -0.14 |
| **SST** | 0.46 | 1.00 | **-0.76** | **0.65** | **-0.75** | **-0.78** | **-0.74** | **-0.76** | **-0.58** | 0.31 |
| Chl | -0.74 | -0.76 | 1.00 | -0.36 | 0.46 | 0.38 | 0.43 | 0.45 | 0.43 | 0.30 |
| Salinity | 0.31 | 0.65 | -0.36 | 1.00 | -0.99 | -0.93 | -0.82 | -0.94 | -0.93 | 0.43 |
| pH | -0.38 | -0.75 | 0.46 | -0.99 | 1.00 | 0.95 | 0.82 | 0.95 | 0.92 | -0.46 |
| DO | -0.17 | -0.78 | 0.38 | -0.93 | 0.95 | 1.00 | 0.93 | 0.97 | 0.77 | -0.51 |
| Nitrate | -0.21 | -0.74 | 0.43 | -0.82 | 0.82 | 0.93 | 1.00 | 0.96 | 0.57 | -0.35 |
| Silicate | -0.31 | -0.76 | 0.45 | -0.94 | 0.95 | 0.97 | 0.96 | 1.00 | 0.77 | -0.41 |
| Iron | -0.40 | -0.58 | 0.43 | -0.93 | 0.92 | 0.77 | 0.57 | 0.77 | 1.00 | -0.27 |
| Phosphate | -0.14 | 0.31 | 0.30 | 0.43 | -0.46 | -0.51 | -0.35 | -0.41 | -0.27 | 1.00 |

Tested for significance, as correlation coefficient (>0.57, < -0.57, p=0.05, 2-tailed)

Correlation of SST with other variables observed significant, represented as bold
