# Supplementary material for: Evaluating the influence of environmental variables on the length-weight relationship and prediction modelling in flathead grey mullet, Mugil cephalus Linnaeus, 1758
Source: PeerJ. 2023 Feb 24;11:e14884. doi: 10.7717/peerj.14884 (PMC9969857; doi:10.7717/peerj.14884)
Supplement: Supplemental Information 2 [file peerj-11-14884-s002.docx]

**Supplemental Table** **2**. Determination of number of factors by Root mean square error (RMSE) & Predicted residual error sum of squares (PRESS) from PLS modelling of response variable and explanatory variables

| Factor | Cross-validation in PLS model | | | | Determination of factors | |
| --- | --- | --- | --- | --- | --- | --- |
|  | RMSE Residuals | PRESS Residuals | Explanatory variables  (% cum Variance) | Response variable  (% cum Variance) | Relative change in RMSE Residuals | Relative change in  PRESS Residuals |
| 1 | 1.08 | -0.28 | 66.4 | 29.1 |  |  |
| 2 | 1.38 | 0.22 | 86.77 | 52.55 | 0.28 | -1.79 |
| **3** | **1.46** | **0.15** | **91.84** | **65.83** | **0.06** | **-0.32** |
| 4 | 1.70 | -0.04 | 93.57 | 88.01 | 0.16 | -1.27 |
| 5 | 1.60 | -0.09 | 99.81 | 90.52 | -0.06 | 1.25 |
| 6 | 20.63 | 0.25 | 100 | 93.11 | 11.89 | -3.78 |
| 7 | 18.08 | -0.08 | 100 | 99.96 | -0.12 | -1.32 |
