# Supplementary material for: Evaluating the influence of environmental variables on the length-weight relationship and prediction modelling in flathead grey mullet, Mugil cephalus Linnaeus, 1758
Source: PeerJ. 2023 Feb 24;11:e14884. doi: 10.7717/peerj.14884 (PMC9969857; doi:10.7717/peerj.14884)
Supplement: Supplemental Information 3 [file peerj-11-14884-s003.docx]

**Supplemental Table** 3. The distribution of climate variable scores (X Scores 1,2,3,) and response variable scores (Y Scores1, 2, 3), distance from model X, Y, predicted weight growth- b(PLS) by 3-factors PLS model, derived from partial least squares (PLS) analysis of weight growth as response variable (Y) and nine climate variables (X)

| Sl. No | Location | Climate variables (X) Scores | | | Response variable (Y)  Scores | | | Distance from model X and model Y | | Outlier | PLS Model  pred. |
| --- | --- | --- | --- | --- | --- | --- | --- | --- | --- | --- | --- |
|  |  | XScore1 | Xscore2 | Xscore3 | Yscore1 | Yscore2 | Yscore3 | D  Mod X | D mod Y | T^2^ (5.44) | b(PLS) |
| 1 | Kakdwip | 0.78 | 0.16 | -0.28 | 1.65 | 0.16 | -0.11 | 0.83 | 0.03 | 5.71 | 2.49 |
| 2 | Paradeep | 0.35 | 0.17 | 0.67 | -0.01 | 0.73 | 0.31 | 1.23 | 0.15 | 4.72 | 3.01 |
| 3 | Chennai | -0.23 | 0.37 | -0.19 | -1.07 | 0.47 | -0.16 | 0.33 | 0.00 | 1.81 | 3.10 |
| 4 | Marakkanam | -0.20 | 0.22 | -0.18 | -0.30 | -0.15 | -0.43 | 0.05 | 0.06 | 0.97 | 3.02 |
| 5 | Puducherry | -0.16 | 0.08 | -0.14 | -0.01 | -0.32 | -0.35 | 0.08 | 0.04 | 0.39 | 2.95 |
| 6 | Cuddalore | -0.20 | 0.20 | -0.22 | -1.46 | 0.90 | 0.39 | 0.06 | 0.37 | 1.03 | 3.00 |
| 7 | Mandapam | -0.31 | -0.03 | 0.36 | -0.39 | -0.30 | -0.19 | 2.50 | 0.31 | 1.85 | 3.14 |
| 8 | Karwar | -0.08 | -0.44 | 0.31 | -1.56 | 1.24 | 1.54 | 0.40 | 1.38 | 2.35 | 2.84 |
| 9 | Ratnagiri | 0.06 | -0.73 | -0.34 | 3.15 | -2.71 | -1.01 | 0.40 | 0.40 | 5.16 | 2.44 |
