# Supplementary figures and images for: Evaluating the influence of environmental variables on the length-weight relationship and prediction modelling in flathead grey mullet, Mugil cephalus Linnaeus, 1758
Source: PeerJ. 2023 Feb 24;11:e14884. doi: 10.7717/peerj.14884 (PMC9969857; doi:10.7717/peerj.14884)

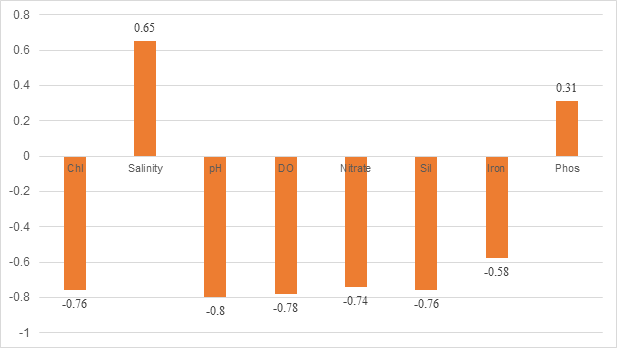

Supplement: Supplemental Information 4 — Chl: Chlorophyll, Sil: Silicate, Phos: Phosphate, DO: Dissolved Oxygen [file peerj-11-14884-s004.png]

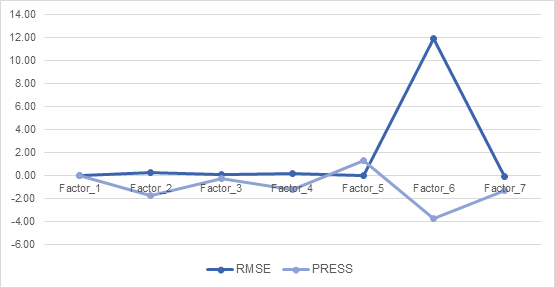

Supplement: Supplemental Information 5 [file peerj-11-14884-s005.png]

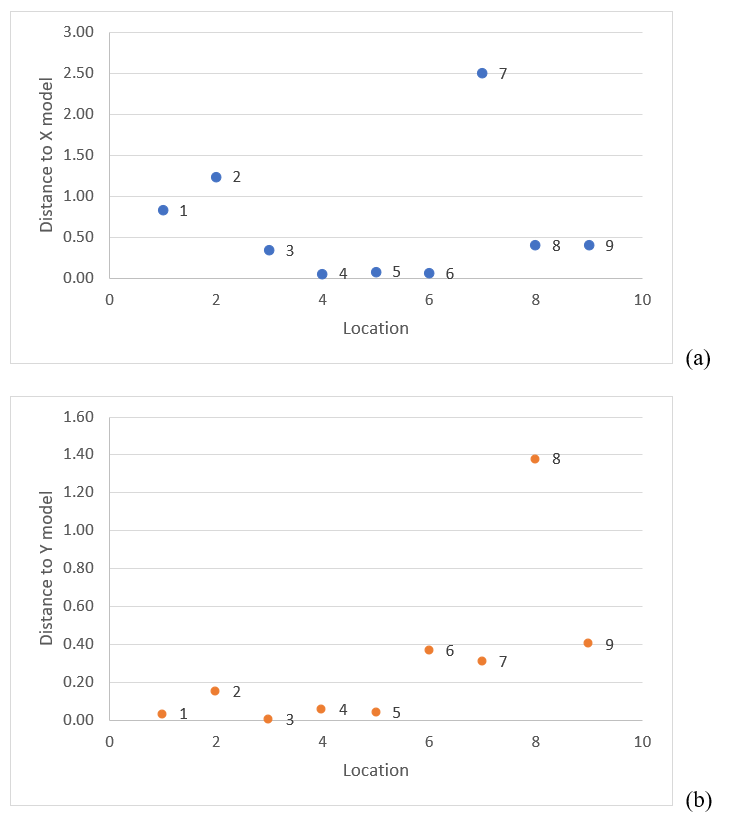

Supplement: Supplemental Information 6 — 1. Kakdwip 2. Paradeep 3. Chennai 4. Marakkanam 5. Puducherry 6. Cuddalore 7. Mandapam 8. Karwar 9. Ratnagiri [file peerj-11-14884-s006.png]

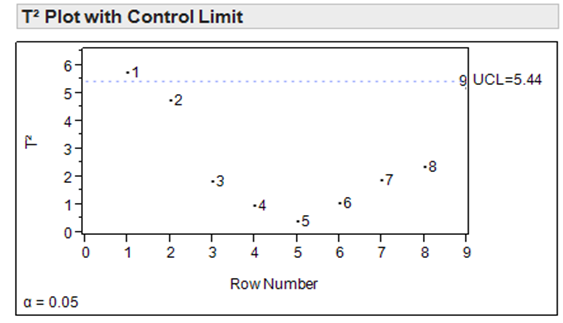

Supplement: Supplemental Information 7 — 1. Kakdwip 2. Paradeep 3. Chennai 4. Marakkanam 5. Puducherry 6. Cuddalore 7. Mandapam 8. Karwar 9. Ratnagiri [file peerj-11-14884-s007.png]

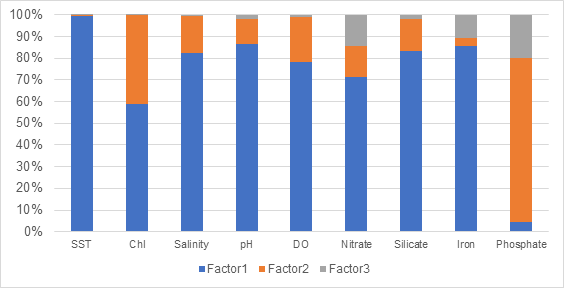

Supplement: Supplemental Information 8 — SST: Sea Surface Temperature, Chl: Chlorophyll, DO: Dissolved Oxygen [file peerj-11-14884-s008.png]

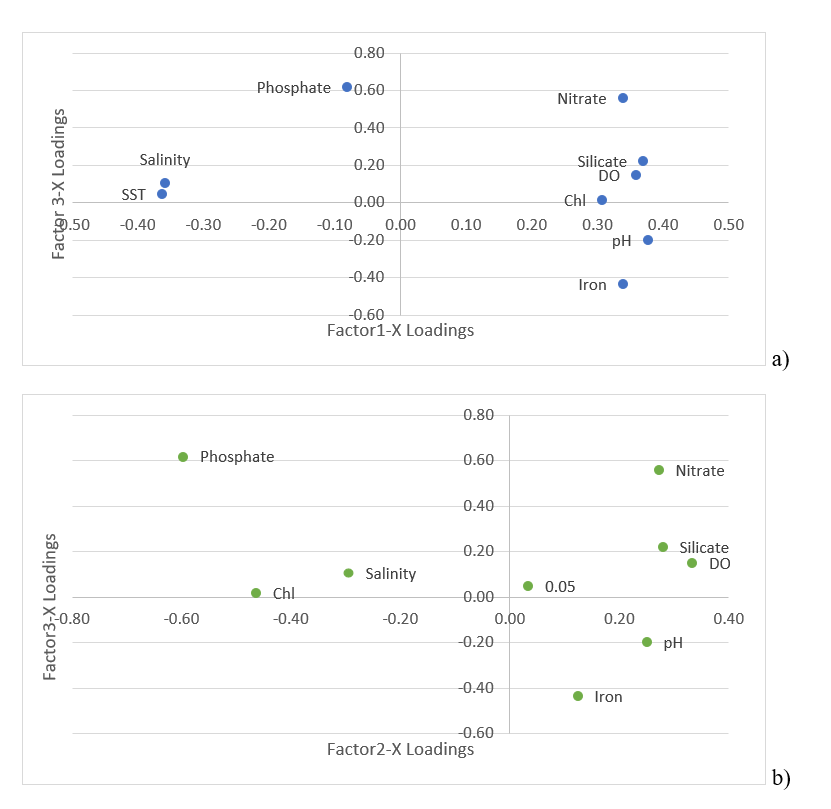

Supplement: Supplemental Information 9 — SST: Sea Surface Temperature, Chl: Chlorophyll, DO: Dissolved Oxygen [file peerj-11-14884-s009.png]

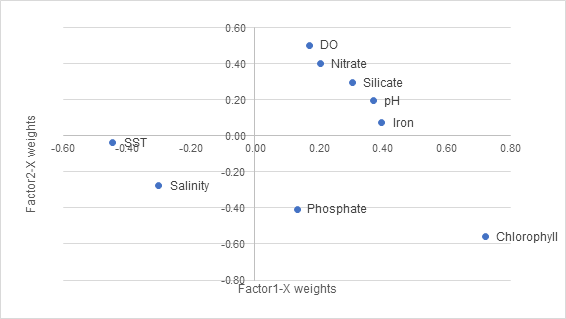

Supplement: Supplemental Information 10 — SST: Sea Surface Temperature, DO; Dissolved Oxygen [file peerj-11-14884-s010.png]

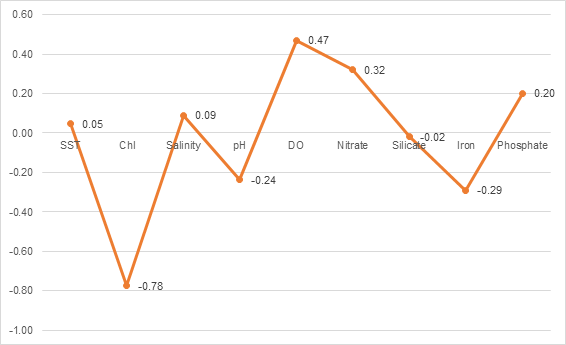

Supplement: Supplemental Information 11 — SST: Sea Surface Temeperature, Chl: Chlorophyll, DO: Dissolved Oxygen [file peerj-11-14884-s011.png]
